# Construction and evaluation of controlled-release delivery system of Abamectin using porous silica nanoparticles as carriers

**DOI:** 10.1186/1556-276X-9-655

**Published:** 2014-12-04

**Authors:** Yan Wang, Haixin Cui, Changjiao Sun, Xiang Zhao, Bo Cui

**Affiliations:** Institute of Environment and Sustainable Development in Agriculture, Chinese Academy of Agricultural Sciences, Beijing, 100081 China

**Keywords:** Abamectin, Silica nanoparticles, Controlled release, Delivery system

## Abstract

Photolysis and poor solubility in water of Abamectin are key issues to be addressed, which causes low bioavailability and residual pollution. In this study, a novel hydrophilic delivery system through loading Abamectin with porous silica nanoparticles (Abam-PSNs) was developed in order to improve the chemical stability, dispersity, and the controlled release of Abamectin. These results suggest that Abam-PSNs can significantly improve the performance of controllable release, photostability, and water solubility of Abamectin by changing the porous structure of silica nanoparticles, which is favorable to improve the bioavailability and reduce the residues of pesticides.

## Background

Nowadays, chemical control is still an important way in protecting crops from biological disasters and reducing the yield loss. Compared with chemical pesticides, biopesticides have attracted increasing attention because of its high bio-efficiency, safety, and environmental friendliness. However, as a typical biopesticide, although Avermectin possesses such advantages over chemical pesticides, its conventional formulations still suffer from some shortages, such as environmental sensitivity, poor water solubility, and short duration and low bioavailability, which limited their large-scale applications in crop production.

The rapid development of nanoscience and nanotechnology provides new ways to improve the performances of conventional pesticide formulations by constructing nanodelivery system using nanomaterials as carriers. In nanomedicine field, many studies suggested that nanoscale carriers can improve the timed release of drug molecules, enable precisive drug targeting, and enhance the drug bioavailability [1–6]. But limited work has been performed on nanoparticle-based pesticide delivery systems. However, the pesticide nanodelivery system has great potential to precisely control the release of pesticide and remarkably reduce effective dosage by maintaining an effective concentration in the target for longer periods of time [7–9], which is favorable to improve the utilization, reduce the residues of pesticides, and avoid the pollution of environment and agricultural products. Moreover, pesticide nanodelivery systems have been proposed to produce a better spatial distribution on leaf surfaces of crops due to the nanoscale size and thereby improve effectiveness of pesticide applications. Besides, pesticide nanodelivery systems also have better penetration through the cuticle and allow slow and controlled release of active ingredients on the target [5, 6].

Silica nanoparticles have attracted many attentions due to their low cost, non-toxicity, high surface area, and high reactivity as drug carriers [10–15]. Although limited works have been done on the pesticide delivery system using silica nanoparticles as carriers [9], some functional defects still need to be improved. Currently, the silica nanoparticles as pesticide carriers did not show controllable porous surface properties, and release of pesticides was generally controlled by adjusting pesticides concentration and the thickness of coating layer, resulting in the relatively limited tuning range of release rate. Moreover, the size uniformity of silica nanocarriers need to be further improved because monodisperse carriers are favorable to promote adhesion and permeability of the pesticide on target crops.

In this study, porous silica nanoparticles (PSNs) with controlled surface properties were employed as novel carriers for loading Abamectin to form Abamectin with porous silica nanoparticles (Abam-PSNs) delivery system. Morphology, pesticide-loading capacity, release rate, and the photolysis of Abamectin were studied for Abam-PSNs delivery system with different porous structures.

## Methods

### Experimental materials

Tetraethylorthosilicate (TEOS), poly(vinylpyrrolidone) (PVP, Mw approximately 10,000), sodium hydroxide (NaOH), and ammonium hydroxide (NH_3_ · H_2_O, 28% by weight in water) were purchased from Sigma-Aldrich (Beijing, China). Ethanol and isopropanol were of analytical grade and obtained from Thermo Fisher Scientific (Beijing, China). All chemicals were directly used as received without further purification.

### Preparation of PSNs and Abam-PSNs

Fifty milliters of a mixture deionized H_2_O (49.5%), NH_3_ · H_2_O (18%), and ethanol (32.5%) was injected into 50 mL of a mixture of TEOS (9%) and ethanol (91%) at room temperature under magnetic stirring. After reacting for 2 h, the silica (SiO_2_) nanoparticles were collected by centrifugation. The prepared SiO_2_ nanoparticles were re-dispersed in deionized water and added with PVP solution (0.1 g/mL) at 100°C to synthesize SiO_2_@PVP nanoparticles. Finally, NaOH solution was added to the SiO_2_@PVP nanoparticles solution as an etching agent and reacted respectively for 45, 75, 105, and 120 min at 30°C to obtain four kinds of PSNs with controllable porous structure. The four kinds of PSNs with different porous structures were used as pesticide carriers to load Abamectin and form Abam-PSNs. In detail, PSNs with different porous structures were mixed with Abamectin in ethyl alcohol solution respectively, and then the mixtures were ultrasonicated for 5 min and vortexed for 24 h in a shaker. Finally, the Abam-PSNs powder was obtained by centrifugation at 13,000 × *g* for 15 min.

### Characterization

The morphologies of the PSNs and Abam-PSNs were investigated by the transmission electron microscopy (TEM, Hitachi-7650, Hitachi Ltd., Chiyoda-ku, Japan) and scanning electron microscope (SEM, JSM-6700 F, JEOL Ltd., Akishima-shi, Japan). The samples of PSNs and Abam-PSNs dispersed in water were cast onto a carbon-coated copper grid, followed by evaporation under vacuum at room temperature for TEM measurement. The prepared PSNs samples were added dropwise onto the silicon slice surface and dried at room temperature for SEM measurement. The specific surface area of the PSNs was measured by Brunauer-Emmett-Teller (BET) method. The pore structure analysis and nitrogen adsorption-desorption isotherms were conducted using a TristarII3020 surface area analyzer (Micromeritics Instrument Corporation, Norcross, GA, USA). The pore size distribution was calculated by Barrett-Joyner-Halenda (BJH) method.

### Investigation on loading performance of Abam-PSNs

The pesticide-loading capacity of Abam-PSNs was determined as follows: the Abam-PSNs suspension were obtained in ethyl alcohol solution after vortexed for 24 h in a shaker according to the method mentioned in the preceding section. The Abamectin concentrations in the supernatant after centrifugation were measured at the wavelength of 245 nm by ultraviolet and visible spectrophotometer (UV-2250, Shimadzu Corporation, Kyoto, Japan). The Abamectin concentrations (C mg/mL) of the suspension were calculated according to their calibration curves. The initial concentration of Abamectin standard solutions is *C*_0_ (mg/mL). Pesticide-loading capacity (PLC) is calculated according to the following formula:


(*V* is the solution volume and *M* is the mass of porous silica carrier).

### Investigation on controlled release behaviors of Abam-PSNs

The release profiles of Abamectin from the Abam-PSNs were investigated as follows: 1 mg Abam-PSNs samples were suspended in 20 mL ethanol solution. The suspension was transferred to the dialysis bag, which were sealed into the 100 mL ethanol solution as the release medium. The release rate of Abamectin from the Abam-PSNs sample was calculated by measuring the concentrations of Abamectin dissolved in the release medium at different times to evaluate the sustained release property. The concentrations of Abamectin were measured by UV-2250 spectrophotometer by collecting 5.0 mL of outside fluid at different intervals of 24, 48, 72, 100, 155, and 200 h. The pure Abamectin (TC) was used as a control.

### Photolysis experiments of Abamectin in Abam-PSNs

The photolytic behavior of Abamectin in the as-prepared Abam-PSNs was evaluated with the pure Abamectin (TC) as a control. The samples were dissolved in ethanol and were irradiated under the light source of an UV fluorescent tube with an emitting light of 365 nm wavelength. The distance of the light source from the upper level of the samples solution was 15 cm. The Abamectin concentration of samples was analyzed at a specified time interval (0, 12, 24, 36, 48, 60, and 72 h).

## Results and discussion

### Preparation and characterization of PSNs

**Figure 1:**
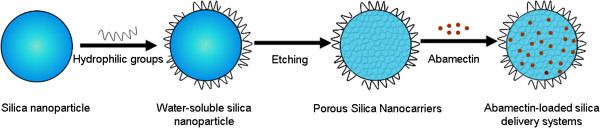
**Graphical presentation of porous silica nanoparticle used as carrier to control the release of Abamectin biopesticide.**

**Figure 2:**
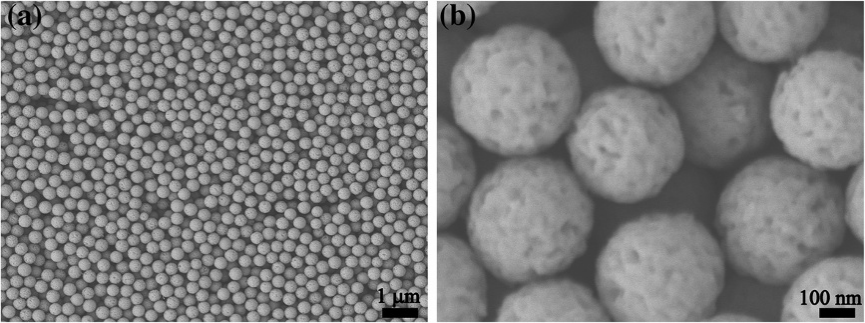
**SEM images of the representative PSNs after etching. (a)** × 10,000 magnification. **(b)** × 100,000 magnification.

**Figure 3:**
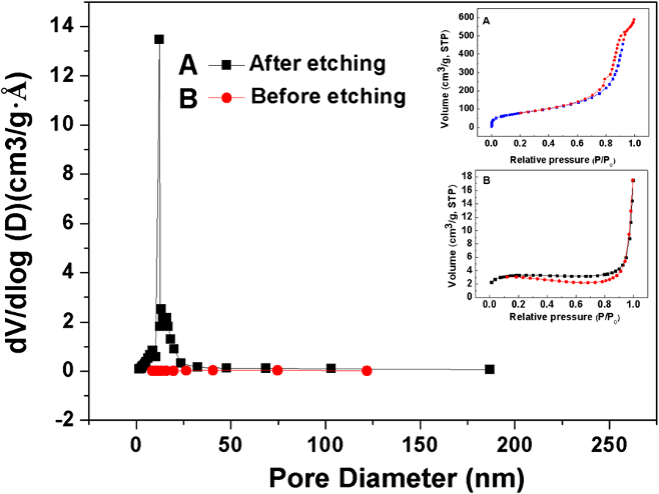
**The nitrogen adsorption-desorption curves and BJH pore size distributions of silica nanoparticles before and after etching.**

Experimental procedures for the construction of Abam-PSNs delivery system were shown in Figure 1. The SEM images of the representative PSNs are shown in Figure 2. The PSNs show uniform spherical shapes with the average diameter of 320 nm. The uniformity and dispersity of the PSNs are favorable to improve the dispersion, adhesion, and permeability of pesticide on target crops as pesticide carriers. SEM imaging confirmed that the surfaces of the PSNs were of obvious porous structures after NaOH etching treatment. The BET results indicated that the specific surface area of the PSNs increased from 11.31 m^2^/g up to 318.62 m^2^/g after etching. The increase of specific surface area can be attributed to gradual etching of the interior and surface layer of silica nanoparticles, which is beneficial to increase the loading capacity of the PSNs as pesticide carriers. The nitrogen adsorption-desorption curves and BJH pore size distributions of silica nanoparticles before and after etching are shown in Figure 3, respectively. The nitrogen adsorption-desorption isotherm of PSNs has different opening and closing pressures before and after etching, indicating the change of pore size in the process of etching. The pore volume of the PSNs remarkably increased after etching, and the pore size distribution of the PSNs was narrow and most of the pores had a diameter of 12 nm.

The results indicated that the PSNs can be successfully synthesized using NaOH as an appropriate etching agent, while carbonyl groups of PVP as the surface protecting agent can form the strong hydrogen bonds with the hydroxyls on the surface of the PSNs. The protection effect of PVP is favorable to increase the stability of silica spheres against collapsion in the etching process and leads to the formation of porous silica nanoparticles. In addition, PVP is favorable to improve pesticide-loading capacity and the water dispersity of the Abam-PSNs.

### Effects of etching time on porous structure of PSNs

**Figure 4:**
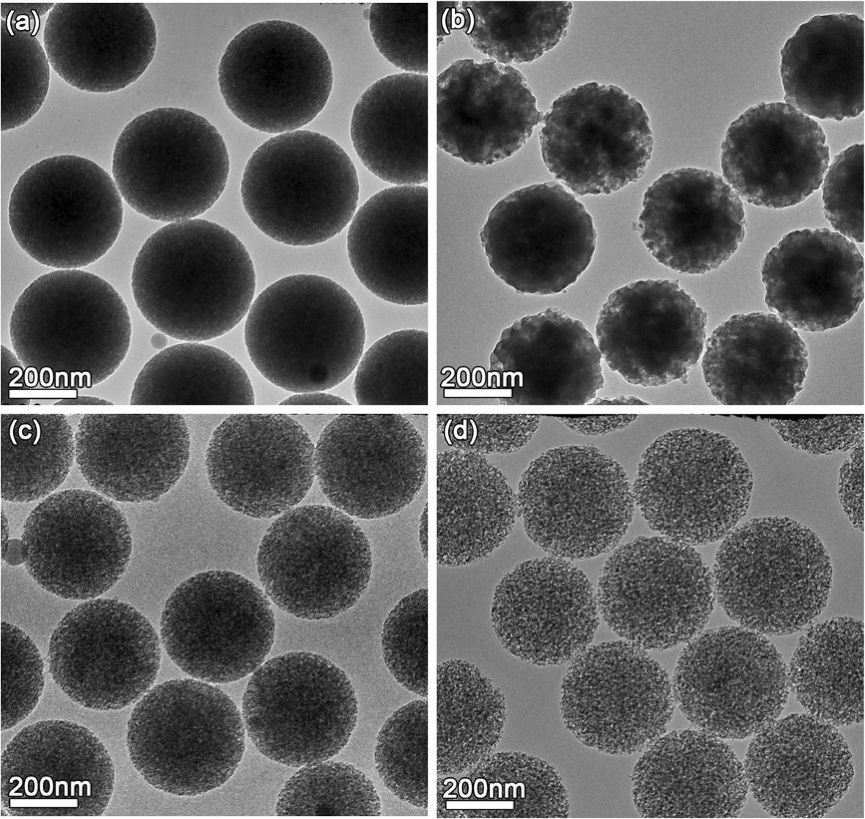
**TEM images of the PSNs etched for different times. (a)** 45 min, **(b)** 75 min, **(c)** 105 min, and **(d)**120 min.

Figure 4 showed TEM images of four kinds of PSNs with different porous structure etched for 45, 75, 105, and 120 min. The imaging results indicated that all of the PSNs have good monodispersity under four different etching conditions, and the porous structure was gradually developed with increased etching time without obvious change of particle size. With the increase of etching time from 45 to 120 min, OH- ions of NaOH can gradually diffuse into the interior of silica nanoparticles and eventually producing porous structures upon continued etching. Continued reaction further makes the appearance of porous structure more pronounced, and the PSNs come to appear rougher and less homogeneous in transmission contrast due to the etching effect. The results suggested that the porous structure of PSNs can be effectively controlled by changing the etching time.

### Effects of porous structure on Abamectin-loading capacity of Abam-PSNs

**Figure 5:**
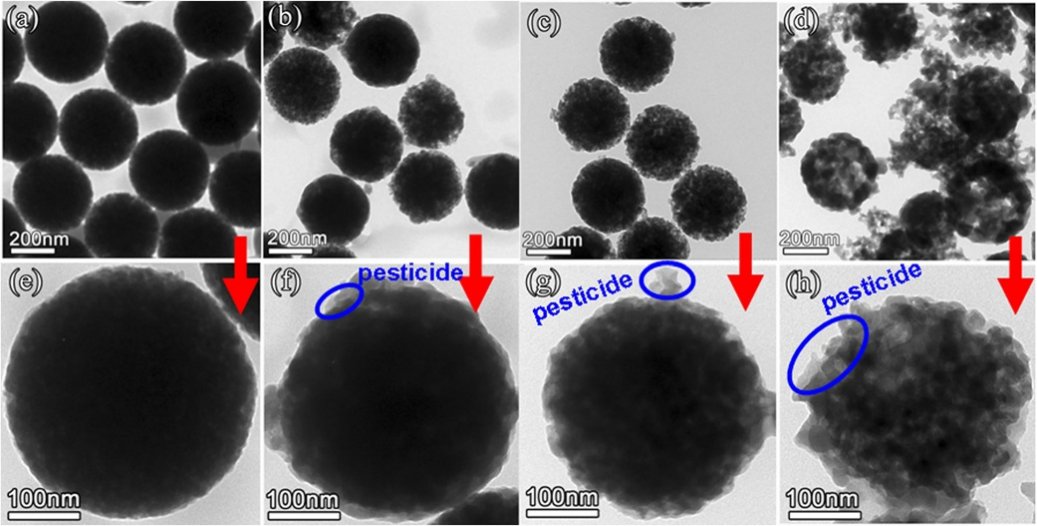
**TEM images of Abam-PSNs with different porous structure obtained at various etching time. (a-d)** At low magnification: **(a)** 45 min, **(b)** 75 min, **(c)** 105 min, and **(d)** 120 min; **(e-h)** the corresponding images of **(a-d)** at high magnification: **(e)** 45 min, **(f)** 75 min, **(g)** 105 min, and **(h)** 120 min.

TEM images in Figure 5 showed the effects of porous structure of PSNs on loading capacity of the Abamectin in Abam-PSNs. The PSNs with different porous structures were used as carriers after etching from 45 to 120 min and load Abamectin to form the Abam-PSNs. The Abam-PSNs show porous and rough surface, which is different with the morphology of PSNs, revealing that the pesticides were adsorbed on the surface of PSNs carriers.

**Figure 6:**
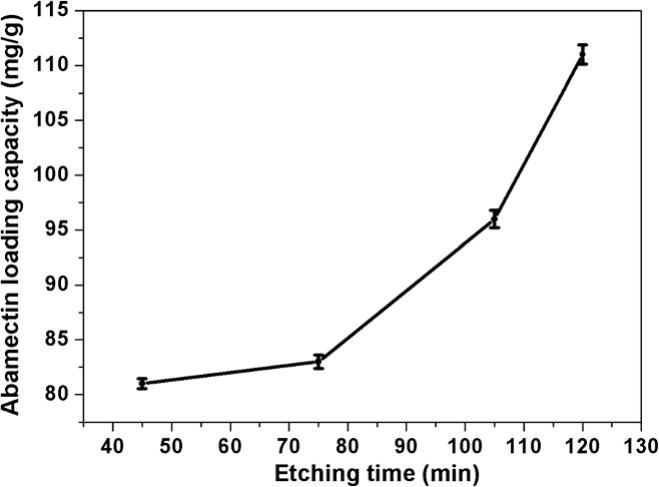
**Abamectin-loading capacity of Abam-PSNs samples formed at different etching times (45, 75, 105, and 120 min).**

The analyzing results of Abamectin-loading capacity of Abam-PSNs formed at different etching times were shown in Figure 6. The Abamectin-loading capacity was increased from 81.0 to 111.0 mg/g with prolonging etching time from 45 to 120 min. The results indicated that Abamectin-loading capacity of Abam-PSNs can be controlled by adjusting the porosity of PSNs carriers.

### Controlled release behaviors of Abam-PSNs

**Figure 7:**
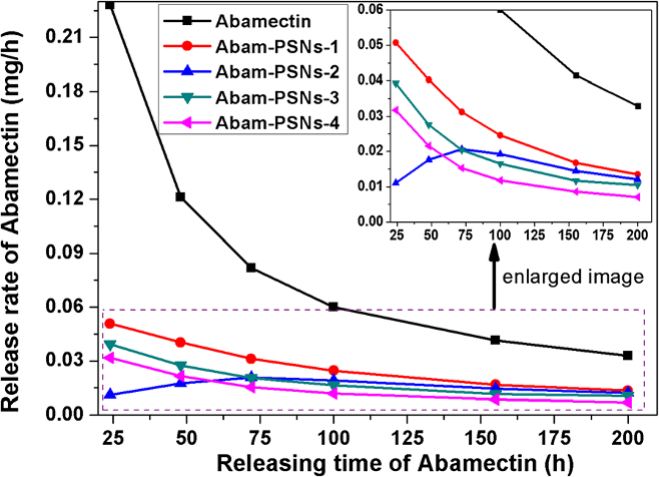
**Release profile of Abamectin loaded by Abam-PSNs with different porous structure.** The pure Abamectin was used as the control in the experiments. Abam-PSNs-1, Abam-PSNs-2, Abam-PSNs-3, and Abam-PSNs-4 samples were etched for 45, 75, 105, and 120 min, respectively.

The release profiles of Abamectin from Abam-PSNs with different porous structures are shown in Figure 7. Compared with pure Abamectin, initial burst release of Abam-PSNs is not so obvious. As expected, Abam-PSNs exhibit slower release rates due to the porous structure on the surface which is favorable to prolong validity period and reduce leaching loss of Abamectin.

The release rate of Abam-PSNs samples was relatively fast at initial stage and then gradually became slow with increased time, as the Abamectin adsorbed on the surface of Abam-PSNs was easier to release than that loaded within the carriers. For the sample with less internally porous structure (Abam-PSNs-1), Abamectin concentrated on the surface nanopores of silica carrier, which make it easier to release pesticide with the fastest release rate than other Abam-PSNs samples. Whereas, for the sample with richer porous structure (Abam-PSNs-3, Abam-PSNs-4), the Abamectin were mainly loaded on the internal porous structure of silica carriers, resulting in longer release time due to relatively slow and smooth release. For the sample of Abam-PSNs-2, the irregular release rate of Abamectin may be due to the mutual interference between fast release on the surface and slow release resulting from decreased surface area which leads to the decreased diffusion gradients and driving force of Abamectin. The results indicated that Abamectin loaded by Abam-PSNs can slowly and controllably be released by adjusting the porous structure of PSNs carriers, resulting in the validity of Abamectin in the target lasting for a longer time. The properties could not only increase the efficiency of pesticide applications and decrease the spraying dosage but also reduce residues and improve the environmental protection.

### Effects of Abam-PSNs on photolysis of Abamectin

**Figure 8:**
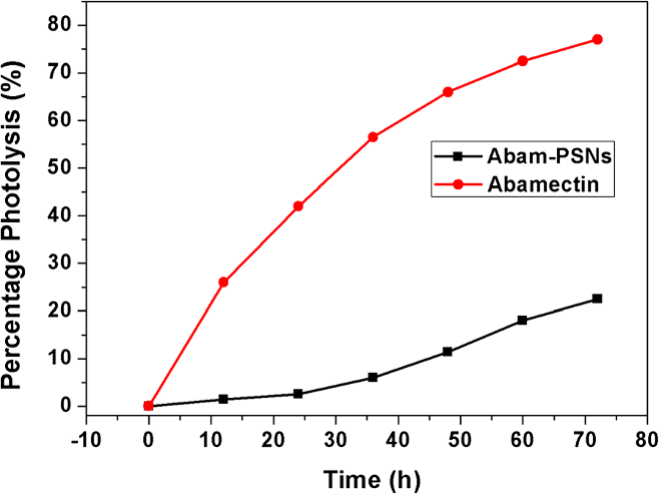
**The response curve of the photolysis rate of pure and loaded Abamectin by Abam-PSNs to UV irradiation time.**

The photolytic rate of Abamectin was estimated by artificial irradiation under UV light. The response curve of the photolysis rate of Abamectin to irradiation time was shown in Figure 8. The photolytic percentage of the Abamectin was 1.4% and 26%, respectively, for the Abam-PSNs and the pure Abamectin after 12 h, while reached 22.5% and 77%, respectively, after 72 h. The results of UV irradiation confirmed that the Abam-PSNs can significantly prevent the photolysis of Abamectin, resulting from the protective effect of the silica carrier.

## Conclusions

In order to improve controlled release, chemical stability, and bioactivity of Abamectin, we developed a nanodelivery system of Abam-PSNs by loading Abamectin with PSNs as carriers. PSNs are synthesized by the process of surface-protected etching, which have developed porous structure controlled by adjusting the etching time. As a novel pesticide carrier, PSNs show excellent pesticide-loading capacity and delivery performance in controlled release, anti-photolysis, and water dispersity. This delivery system is favorable to overcome the shortages of biopesticides, such as environmental sensitivity, poor water solubility, and hence improve efficacy in crops protection, decreased the spraying dosage, and reduce residues and pollution in food and environment.

## Authors’ information

YW is an assistant professor, HC is a professor, CS and BC are assistant professors, and XZ is a graduate student in the Institute of Environment and Sustainable Development in Agriculture, Chinese Academy of Agricultural Sciences.
